# Chronic nicotine pretreatment is sufficient to upregulate α4* nicotinic receptors and increase oral nicotine self-administration in mice

**DOI:** 10.1186/1471-2202-15-89

**Published:** 2014-07-19

**Authors:** Anthony Renda, Raad Nashmi

**Affiliations:** Department of Biology, University of Victoria, PO Box 3020, Station CSC, Victoria, BC V8W 3 N5 Canada

## Abstract

**Background:**

Understanding the underlying causes of nicotine addiction will require a multidisciplinary approach examining the key molecular, cellular and neuronal circuit functional changes that drive escalating levels of nicotine self-administration. In this study, we examined whether mice pretreated with chronic nicotine, at a dosing regimen that results in maximal nicotinic acetylcholine receptor (nAChR) upregulation, would display evidence of nicotine-dependent behaviour during nicotine self-administration.

**Results:**

We investigated oral self-administration of nicotine using a two-bottle choice paradigm in which one bottle contained the vehicle (saccharine-sweetened water), while the other contained nicotine (200 μg/ml) in vehicle. Knock-in mice with YFP-tagged α4 nAChR subunits (α4YFP) were implanted with osmotic pumps delivering either nicotine (2 mg/kg/hr) or saline for 10 days. After 10 days of pretreatment, mice were exposed to the nicotine self-administration paradigm, consisting of four days of choice followed by three days of nicotine abstinence repeated for five weeks. Mice pre-exposed to nicotine had upregulated α4YFP nAChR subunits in the hippocampal medial perforant path and on ventral tegmental area GABAergic neurons as compared to chronic saline mice. Compared to control saline-pretreated mice, in a two bottle-choice experiment, nicotine-primed mice ingested a significantly larger daily dose of nicotine and also exhibited post-abstinence binge drinking of nicotine.

**Conclusions:**

Chronic forced pre-exposure of nicotine is sufficient to induce elevated oral nicotine intake and supports the postulate that nAChR upregulation may be a key factor influencing nicotine self-administration.

## Background

Tobacco smoking inflicts a heavy toll on the world’s population, cutting the life expectancy of smokers by up to ten years [1] and nicotine dependence is the primary reason for the continued use of tobacco even in the face of compromising one’s health. The behaviours of drug seeking, reinforcement, tolerance and withdrawal associated with nicotine addiction are mediated at the molecular level by nicotinic acetylcholine receptors (nAChRs) [2–5].

Nicotine targets many areas in the brain due to the fact that nAChRs have a widespread distribution throughout the CNS [6, 7]. There are a total of 12 neuronal subunits in vertebrates (α2-10, β2-4) assembling into a variety of nAChR subtypes, of which the major subtype in the brain is the heteropentameric α4β2* nAChR (* denotes that the receptor contains other subunits in addition to α4 and β2) [8]. Nicotine’s impact on various aspects of nicotine addictive behavior, including drug seeking, reinforcement, tolerance and withdrawal is likely due to nicotine targeting nAChRs localized on different neuronal circuits in the brain. The ventral tegmental area is a key neural substrate located in the ventral midbrain which mediates reward, reward prediction or even aversion [9–11] and is therefore a prime target for drugs of abuse such as nicotine [4, 12–14].

Nicotine provides positive reinforcement by activating α4-containing (α4*) and β2-containing (β2*) nAChRs in the ventral tegmental area [3–5, 14, 15] and chronic administration of nicotine upregulates high-affinity α4β2 nAChR expression in mice [6, 7, 16] and rats [17], altering the molecular circuitry that governs reward. Importantly, the brains of human smokers also show upregulation of nicotinic receptors [18, 19], a key biochemical hallmark of smoking. Nicotinic receptor upregulation occurs in a brain region and cell-type specific manner, although it is unclear whether nAChR upregulation is merely an epiphenomenon or an actual contributor to addictive behaviour. However, upregulation of α4* nAChRs on GABAergic neurons of the VTA is implicated in behaviours of tolerance and craving of nicotine in chronically exposed mice [7]. With regards to drug taking, craving induces a cycle of administration and reinforcement to perpetuate drug seeking, while tolerance causes heightened administration. Abstinence from nicotine causes withdrawal, characterized by both somatic illness [2, 20–22] and cognitive deficits [23–27]. Severity of withdrawal also correlates with nicotinic receptor upregulation [28] and symptoms are attenuated by injection of nicotine [21]. Specifically, β2* nAChRs and nAChR upregulation have been shown to be associated with cognitive withdrawal [24, 27]. However, some studies reported that nAChR upregulation is not correlated with physical withdrawal symptoms but instead that physical withdrawal depends on nAChRs containing α2, α5 and β4 subunits and not β2 subunits [2, 22]. Furthermore, physical symptoms of withdrawal appear to be mediated by brain regions outside the VTA, including the medial habenula – interpeduncular nucleus pathway [22, 29]. Thus, there are some conflicting data concerning whether nAChR upregulation correlates with withdrawal, but little is known about whether upregulation contributes to heightened nicotine self-administration.

Oral self-administration, using drinking water as the vehicle for drug administration, is a well established paradigm for nicotine self-administration [16, 30, 31]. This method is non-invasive and can also provide adequate means to study the effects of nicotine intake in an episodic manner and during periods following overnight abstinence, paralleling that of compulsive tobacco users. In this study, using a two bottle-choice of oral nicotine self-administration, we tested the hypothesis that mice chronically pretreated with nicotine and having upregulated nAChRs will self-administer more nicotine than mice that have normal receptor levels. We also examined whether nicotine abstinence will perpetuate elevated nicotine self-administration.

## Methods

### Mice

All experiments were conducted in accordance with the guidelines for care and use of animals provided by the Canadian Council on Animal Care Use. Mice were housed at the University of Victoria Animal Care Unit and all protocols were approved by the University of Victoria Animal Care Committee. Eight to 10 week old homozygous male α4YFP knock-in mice were used in all experiments [7, 32]. In the α4YFP knock-in mouse strain its α4 nAChR gene was altered so that the coding cDNA sequence of the yellow fluorescent protein (YFP) was inserted into the M3-M4 loop of the α4 nAChR subunit. The α4YFP* nAChRs function and express normally in every respect [7]. The strain is back-crossed over 10 generations to C57BL/6 J. Mice were housed on a 12 hour light/dark cycle at 22°C and given a standard laboratory diet and water *ad libitum*, except where noted in behavioural experiments.

### Chronic nicotine administration via osmotic pumps

Chronic nicotine or saline was administered via implanted osmotic pumps (model 2002; Alzet, cat# 7147090–12) with a flow rate of 0.5 μl/h. Solutions, either nicotine or saline-control, were prepared on the day of surgery and stored in saline before implantation. Control pumps were filled with saline (0.9% w/v, cat# S5815, Teknova). Nicotine-containing pumps were filled with (−)-nicotine hydrogen tartrate (cat# N5260, Sigma) at a concentration to deliver nicotine at 0.4 mg/kg/hr or 2 mg/kg/hr (calculated as free base of nicotine) for 10 days. Surgery was performed as described in Renda and Nashmi [32]. The 10 day duration and amount of nicotine delivery (2 mg/kg/h) via this method is sufficient to cause maximal nAChR upregulation [33] and a blood nicotine concentration of ~590 nM [34] which is near the peak concentration of nicotine found in smokers’ blood.

### Fixation and brain sectioning

Following 10 days of chronic nicotine administration via osmotic pumps, mice were sacrificed and their brains harvested for quantitative fluorescence imaging. Mice were anesthetized via intramuscular injection of ketamine (100 mg/ml) and dexmedetomidine hydrochloride (0.5 mg/ml) and intracardially perfused with 20 ml of PBS (pH 7,6), followed by 30 ml of 4% paraformaldehyde in PBS (pH 7.6, cat# 15710, Electron Microscopy Sciences) and finally with 5% sucrose in PBS (pH 7.6) in order to flush residual PFA and reduce autofluorescence. The brain was then extracted and placed in 30% sucrose in PBS (pH 7.6). After three days the brains were removed, submerged in O.C.T. Mounting Compound (cat# 4583, Tissue-Tek), frozen in dry ice and then stored at −20°C before slicing. Brains were sectioned coronally (30 μm thick) on a cryostat (Leica CM1860UV), transferred to coated slides (cat# 15-188-48, Superfrost® Plus Gold, Fisher Scientific) kept in slide boxes containing a calcium sulphate stone to prevent freezer burn and stored in zip-lock bags at −20°C.

### Immunohistochemistry

Brain sections on slides were washed twice with PBS (pH 7.6) for 10 min, and then permeabilized with 0.25% Triton for 5 min. The slides were washed twice again for 10 min with PBS, then blocked with 10% donkey serum in PBS (cat# 017-000-121, Jackson ImmunoResearch) for 30 min. The primary antibodies (tyrosine-hydroxylase polyclonal antibody, cat# P4010-0, Pel-Freez; GAD67 monoclonal antibody, cat# MAB5406, Millipore) were diluted in 3% donkey serum in PBS at a 1:100 concentration and incubated for 60 min at 37°C. Slides were washed with PBS three times for 5 min. The secondary antibodies (Alexa Fluor 405 IgG secondary antibody, cat# A-31556, Invitrogen; Cy5 IgG secondary antibody, cat# 715-175-150, Jackson ImmunoResearch Labs) were diluted in 3% donkey serum in PBS, at a 1:200 concentration and incubated for 60 min at 37°C. Brain slices were then washed with PBS for 5 min three times. Brain sections were mounted with 25 μl Vectashield Mounting Medium (cat# H-1000, Vector Laboratories) and coverslipped.

### Spectral confocal imaging

Image collection was performed as described in detail by Renda and Nashmi [32]. Images were acquired using a Nikon C1si spectral confocal system attached to a Nikon Eclipse Ti-E inverted microscope. Samples were imaged using a 60X oil CFI Plan Apo VC objective (1.40 NA, 0.13 mm working distance) and α4YFP was excited using a 488 nm laser line of a 40 mW Argon laser at 15% maximum transmission, imaged at 4.08 μs pixel dwell time and averaged over two scans through a 60 μm diameter pinhole with spectral detector gain at 220. Alexa 405 secondary antibody was imaged with a 405 nm diode laser at 3.5% transmission and CY5 conjugated secondary antibody labeling was imaged with a 638 nm diode laser at 7% transmission. We collected 50 μm × 50 μm z-stack images through 30 μm thick tissue over a spectral range of 496.5 nm - 656.5 nm at 5 nm resolution. Thus a λ-stack of images were acquired at 512 × 512 pixels. Hence, each pixel of the X-Y image is comprised of a complete spectral emission profile. The endogenous expression of the α4YFP fluorophore in our mouse line allowed us to image the tissue and avoid the confounds of antibody specificity and penetration [32]. Settings remained constant during imaging of all brain sections and for each laser line the laser power were checked with a power meter at the beginning of each day’s experiment. A linear unmixing algorithm was used to deconvolve specific YFP fluorescence from background autofluorescence based on the unique spectral signatures of YFP imaged from soluble YFP transfected HEK293T cells and brain regions from WT C57BL/6 J mice. Once separated, autofluorescence is discarded and the remaining image displaying values only pertaining to YFP expression remains. Details of spectral unmixing are provided in Renda and Nashmi [32].

### Two bottle-choice nicotine self-administration

**Figure 1:**
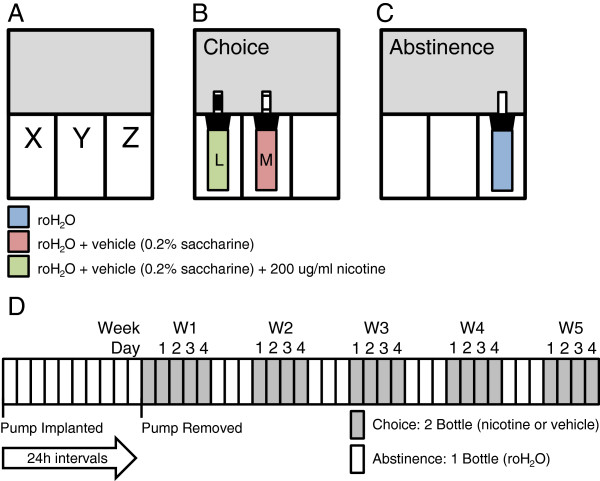
**Two bottle-choice administration schematic and time frame. A)** The wire cage top can be divided into three sections (X | Y | Z). **B)** During periods of choice mice are presented with bottles in X and Y, both of which contain roH_2_O and the vehicle: 0.2% saccharine. One bottle will contain 200 μg/ml nicotine (green) and the second will contain only vehicle (red). Lateral (X) or medial (Y) placement of the nicotine-containing bottle is randomized. The spout of each bottle is randomly labeled black or white as a visual cue to reduce nicotine consumption due to taste-testing. Food is placed in the unoccupied third of the wire cage top, Z. **C)** During abstinence, the mice are given a single bottle containing only roH_2_O (blue). It is placed in Z and the food is moved to X | Y. The spout of the abstinence bottle is unlabeled. **D)** Mice are implanted with osmotic pumps containing nicotine or saline, for ten days, with access to a standard laboratory diet and water. On day ten the pump is removed and the mice are subjected to periods of two bottle-choice (grey: 0.2% saccharine in roH_2_O containing either nicotine or vehicle), followed by 3 days of abstinence (white: 1 bottle containing roH_2_O). Cycles of choice and withdrawal continue for an additional 4 weeks.

Individually housed α4YFP mice were implanted with either saline or nicotine-containing osmotic pumps and given standard food and water *ad libitum* for 10 days, as above. On day 10 the pumps were removed via the initial incision site. Mice were anesthetized in order to remove the pumps in the same manner as described above for implantation. Two choices of drinking water (reverse osmosis water (roH_2_O)) were then provided for 5 days, one of which contained 200 μg/ml (−)-nicotine (Sigma, cat# N3876). Bottles were constructed from 50 ml plastic centrifuge tubes with rubber stoppers that were fitted with metal spouts. A cage with no mice but with two filled bottles were monitored daily to control for fluid loss due to fluid evaporation and spillage. Both bottles were sweetened with a vehicle: 0.2% saccharine (Sigma-Aldrich, cat# 109185). The spout of each bottle was labeled with either white or black tape to indicate which solution (nicotine or vehicle) was present in order to reduce drinking variability due to the animal trying to determine the contents of each bottle. This was randomized so that an equal number of mice were drinking nicotine labeled with black tape as white tape within the saline pump group of mice and within the nicotine pump group of mice. The two choice-bottles were placed beside each other, with food occupying the remaining portion of the wire cage-top. We randomized the placement of the nicotine bottle for each mouse and ensured an equal number of nicotine bottles in the lateral vs middle position within each group (nicotine or saline) to avoid any potential mouse bias for bottle position. Once spout color and bottle position were assigned to the nicotine and vehicle bottles, the bottle position and spout color assignment remained the same throughout the experiment for each mouse. The spout color and bottle position were cues that the mouse could associate whether the bottle contained nicotine or saccharine water. The rationale is that predictive cues have been shown to be effective in enhancing nicotine self-administration in rodents [35]. After five days of choice, the nicotine and vehicle bottles were removed and replaced with an identical bottle with an untaped spout containing only roH_2_O in order to induce withdrawal from nicotine. After three days of abstinence, the mice were put back on the two bottle-choice setup for four days, followed again by three days of abstinence. This weekly cycle continued for five total periods of choice, including the first period following pump removal. Mice were weighed on the second day of each abstinence period to minimize the effects of stress on their nicotine consumption. Bottles were weighed each day and replaced with fresh solutions. Nicotine consumption was recorded every 24 h as a daily dose (mg nicotine/kg mouse) and the mouse weight used in the dose calculation was an average of the weight measurements taken during the abstinence period prior to and following the period of choice in question. Nicotine consumption was also recorded as the amount of fluid consumed from the bottle containing nicotine as a percentage of the animals’ total daily fluid intake. Note that during the day of pump removal surgery, even though mice were presented with a choice of two bottles, drinking volumes were not included in the results to allow mice one day of recovery from surgery (Figure 1).

### Statistical analysis

All values are reported as mean ± standard error of the mean (SEM). Statistics were performed using the R Project for Statistical Computing language (http://www.r-project.org). To compare statistical difference between two groups of data a t-test was performed provided the data met parametric requirements of normality (Shapiro-Wilk normality test) and equal variance (Fligner-Killeen test of homogeneity of variances). Otherwise, the non-parametric equivalent, the Wilcoxon rank sum test, was performed. For comparison of three or more groups a one-way analysis of variance (ANOVA) was used for parametric data and a Kruskal-Wallis rank sum test was used for non-parametric data. A two-way ANOVA was performed on data with two factor variables. A Bonferroni correction was performed as a post-hoc pair-wise analysis on data deemed significant by either one-way or two-way ANOVA. A Wilcoxon rank sum test was used as post-hoc pair-wise analysis on data deemed significant using the Kruskal-Wallis rank sum test. Groups of data were calculated to be statistically different from each other at p < 0.05 level.

## Results

### Chronic nicotine administered via pumps upregulates α4YFP nAChRs

Nicotinic receptor upregulation is a biochemical hallmark in the brains of smokers and chronic nicotine infusion in mice can upregulate nAChRs in various brain regions. However, it is unclear whether nAChR upregulation plays any role in nicotine addiction. We hypothesized that mice pretreated with chronic nicotine at a dosing regimen sufficient to upregulate nicotinic receptors would self-administer a higher dose of nicotine as compared to control mice, which receive a pretreatment of chronic saline. Hence, we looked for evidence of an addictive behaviour that would correlate with nicotine pretreatment and receptor upregulation. To test our hypothesis, we pretreated mice with chronic nicotine via an osmotic pump implanted subcutaneously that delivered nicotine at 2 mg/kg/hr for 10 days. We first confirmed that the chronic nicotine exposure via the pump at our dosing regimen caused upregulation of α4* nAChRs in specific brain regions.

**Figure 2:**
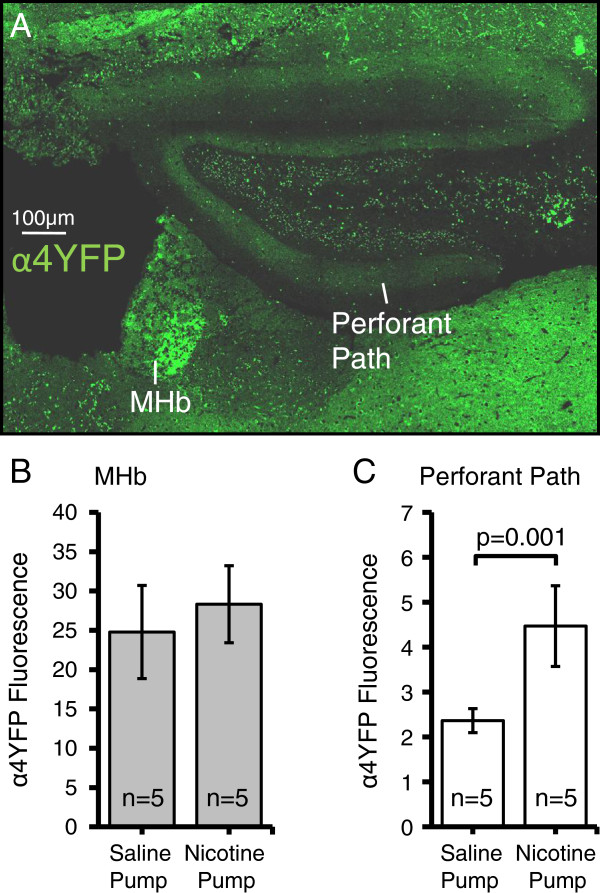
**Changes in α4YFP nAChR expression in the medial habenula and the hippocampal medial perforant path. A)** Montage of images of α4YFP nAChR expression in the medial perforant path and medial habenula (MHb) in a coronal mouse brain section. **B)** Chronic nicotine does not affect α4YFP nAChR expression in the MHb (14 ± 17% upregulation, p = 0.3, t-test). **C)** Chronic nicotine results in upregulation of α4YFP nAChRs in the medial perforant path (89 ± 16% upregulation, p = 0.001, Wilcoxon rank sum test) in mice.

Previously, we and others have shown that chronic nicotine delivery results in a region specific upregulation of α4* nAChRs [6, 7, 16, 17]. Specifically, we demonstrated that 10 days of chronic nicotine delivery at 2 mg/kg/hr caused the largest increase in α4* nAChR expression in the medial perforant path of the hippocampus (94 ± 2%), whereas the medial habenula (MHb) was unaffected by the drug (12 ± 4%) [7]. Thus, the medial perforant path and the MHb are used as positive and negative controls, respectively, to examine nicotine induced α4YFP upregulation. We have replicated those results, with chronic nicotine causing an 89 ± 16% upregulation of α4YFP in the medial perforant path (p = 0.001, Wilcoxon rank-sum test) while causing no significant α4YFP nAChR upregulation in the MHb (14 ± 17% upregulation, p = 0.3, two sample t-test) (Figure 2C,D) when compared to control mice in which osmotic pumps delivered saline for 10 days.

**Figure 3:**
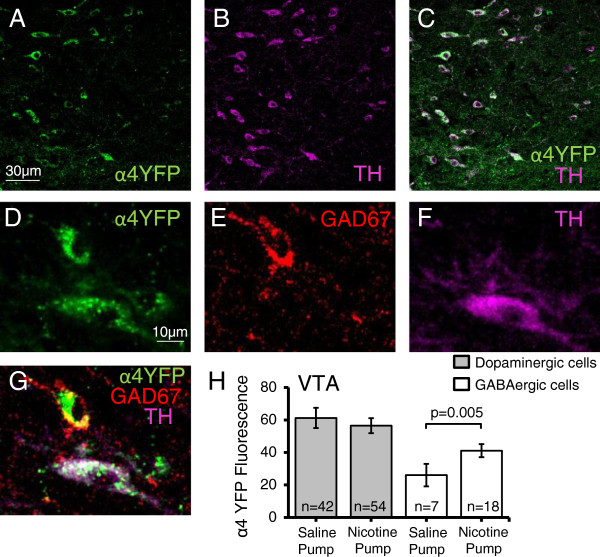
Chronic nicotine increases expression of α4* nAChRs on GABAergic somata of the ventral tegmental area (VTA). A-C) Low magnification images showing α4YFP expression (green) in dopaminergic neurons (TH, magenta) in the VTA. **D-F)** Higher power images showing immunohistochemical labeling of α4YFP (green) **(D)** on GABAergic (GAD67, red) **(E)** and dopaminergic (TH, magenta) **(F)** neurons of the VTA. **G)** Merged image showing α4YFP expression on a TH positive and a GAD67 positive neurons. **H)** Chronic nicotine causes an upregulation (57 ± 10%) of α4YFP* nAChRs on GABAergic somata of the VTA (p = 0.005, two sample t-test) as compared to saline controls, but α4YFP expression in dopaminergic neurons are unaffected (−8 ± 8%) (p = 0.5, Wilcoxon rank sum test). All n values are the total number of neurons imaged and analyzed from 5 nicotine pump mice and 5 saline pump mice.

Nashmi and colleagues [7] were the first to investigate cell-type specific changes of neuronal nAChRs in the brain. We previously showed that chronic nicotine causes a 36 ± 7% receptor upregulation in ventral tegmental area (VTA) GABAergic neurons but no upregulation on dopaminergic soma. Here, we measured the mean α4YFP fluorescence in each individual GABAergic and dopaminergic cell body by co-immunostaining for GAD67 and tyrosine hydroxylase, respectively. We showed similar results to the previous study: chronic nicotine caused a 57 ± 10% upregulation on GABAergic somata (p = 0.005, t-test) but no upregulation (−8 ± 8%) on dopaminergic somata (p = 0.5, Wilcoxon rank sum test) of the VTA when examined from Bregma −2.92 mm to −3.16 mm (Figure 3).

Imaging the brains of α4YFP mice exposed to chronic nicotine or chronic saline via osmotic pumps and quantifying fluorescence yielded cell specific patterns of α4YFP nAChR upregulation in specific brain regions. Table 1 compares our fluorescence intensity values of α4YFP expression following chronic nicotine exposure as a percentage of α4YFP expression in nicotine naïve mice (saline pump), with those obtained by Nashmi et al. [7]. This study was successful in replicating receptor expression level changes, following exposure to chronic nicotine, in specific brain regions and neuronal cell types as in Nashmi et al. [7]. Furthermore, the magnitude of the percentage of receptor upregulation in different brain regions was nearly identical in both studies. Therefore, these data validate the quantification of α4* nAChRs performed in this study.

**Table 1:** Changes in α4YFP* nAChR expression with chronic nicotine exposure in α4YFP knock-in mice in this study as compared to Nashmi et al. [7]

		α4YFP* nAChR Upregulation (% of saline)	
Brain Region	Cell Type	This study	Nashmi et al. [7]
Perforant Path	Glutamatergic	189 ± 16 (p = 0.001)	194 ± 2 (p < 0.001)
Medial Habenula	Cholinergic	114 ± 17 (ns)	112 ± 4 (ns)
VTA	GABAergic	157 ± 10 (p = 0.005)	136 ± 7 (p = 0.002)
	Dopaminergic	92 ± 8 (ns)	109 ± 3 (ns)

ns = not significantly different from saline treated animals.

### Effect of two bottle-choice nicotine self-administration on bottle position preference, animal weight and daily fluid intake

**Figure 4:**
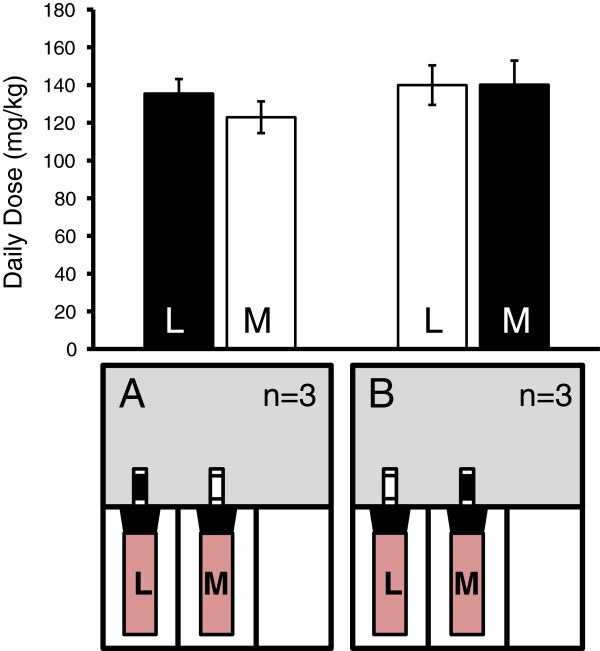
**Mice showed no preference for bottle position or spout colour.** Control mice (n = 3 for each setup A and B) were implanted with saline-containing pumps for 10 days and then subjected to the two bottle-choice paradigm. Both bottles contained only vehicle (roH_2_O sweetened with 0.2% saccharine (red)) and the spout was labeled either black or white, as depicted in A and B. Bottles were randomized so that half the mice had their laterally positioned bottle (L) labeled black and half had their medial positioned bottle (M) labeled black. Neither group showed any preference for bottle position or spout color (A: p = 0.13, Wilcoxon rank sum test, n = 3; B: p = 0.60, Wilcoxon rank sum test, n = 3).

To ensure bottle preference was not biased towards spout colour or bottle location, six mice were implanted with pumps containing saline for 10 days and then subjected to the two bottle-choice paradigm (Figure 1). During periods of choice each bottle contained only vehicle (roH_2_O + 0.2% saccharine), one with a white spout and one labeled black. Positions of the bottles were randomized so that half the mice (n = 3) had their laterally positioned bottle labeled black while the other half (n = 3) had their medial bottle labeled black. Regardless, neither group showed any preference for bottle position (lateral or medial) nor spout colour (black or white) (lateral black spout: p = 0.13, Wilcoxon rank sum test; medial black spout: p = 0.60, Wilcoxon rank sum test) (Figure 4).

**Figure 5:**
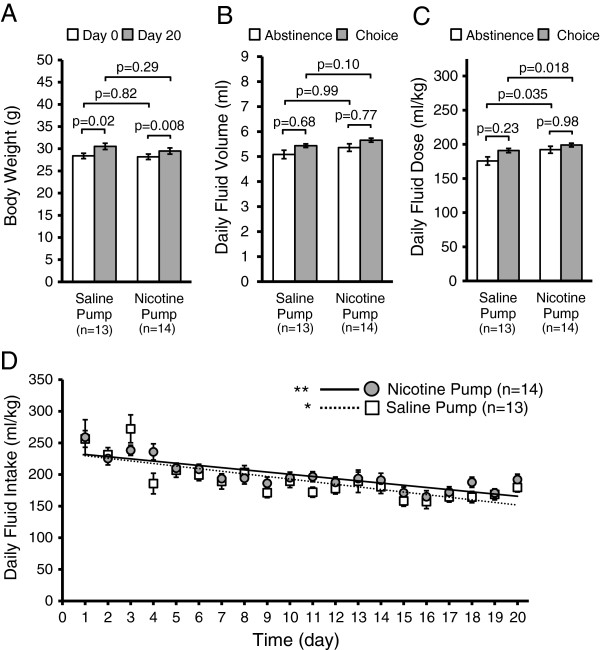
**Effect of a two bottle-choice nicotine self-administration assay on bottle preference, animal weight and daily fluid intake. A)** Mice in both groups that were either implanted with saline pumps or nicotine pumps displayed significant increase in body weight from day 0 to day 20 over the course of the nicotine self-administration experiment. However, there was no significant difference in body weight between saline pump and nicotine pump mice either on day 0 or on day 20 of the experiment. **B)** There was no difference in daily fluid volume (totaled for both bottles, ml) consumed between the choice or abstinence periods in either the saline pump mice or the nicotine pump mice. Furthermore, there was no difference in fluid volumes consumed between saline pump mice and nicotine pump mice during choice or abstinence. **C)** Daily fluid intake normalized to animal body weight (totaled for both bottles, ml fluid/kg mouse) showed that nicotine pump mice consumed a significantly larger daily total fluid dose compared to saline pump mice during periods of choice and also during periods of abstinence, although neither the nicotine pump mice nor the saline pump mice consumed more fluid during choice as compared to during abstinence. **D)** Both the saline pump and nicotine pump mice showed a linear trend of decreased total fluid intake over the 20 days of the self-administration experiment (saline pump: dashed line, R^2^ = 0.61, *p = 0.0003, linear regression) (nicotine pump: solid line, R^2^ = 0.70, **p < 0.0001, linear regression).

The initial weights of each group of mice were not significantly different (Day 0, p = 0.82, two sample t-test with Bonferroni correction), and although both groups gained a significant amount of weight over the test (saline pump, p = 0.02, n = 13; nicotine pump, p = 0.008, n = 14, paired t-tests with Bonferroni correction), the animals’ weights on the final day of self-administration was not different, indicating that neither nicotine pump mice nor saline pump mice had their body mass differentially affected by nicotine over the course of self-administration (p = 0.29, two sample t-test with Bonferroni correction) (Figure 5A). Fluid consumption was very similar for both nicotine and saline pump mice (Figure 5B,C). There was no difference in daily fluid volume consumed between saline pump and nicotine pump mice during choice (p = 0.10) or abstinence (p = 0.99, Wilcoxon rank sum tests with Bonferroni correction). Neither did either group consume different fluid volumes during choice or abstinence (saline pump, p = 0.68; nicotine pump, p = 0.77, Wilcoxon rank sum tests with Bonferroni correction) (Figure 5B). However, once the slight differences in body weight are taken into account, we see that nicotine pump mice consumed a significantly larger daily total fluid dose normalized to body weight (ml/kg) as compared to saline pump mice during periods of choice (p = 0.018) and abstinence (p = 0.035), although neither the saline pump mice nor the nicotine pump mice consumed more fluid during choice vs abstinence (saline pump, p = 0.23; nicotine pump, p = 0.98, Wilcoxon rank sum tests with Bonferroni correction) (Figure 5C). Both groups of mice, nicotine pump and saline pump, steadily decreased their total volume of fluid intake over the course of the two-bottle choice experiment from day 1 to day 20 (nicotine pump: R^2^ = 0.70, p < 0.0001, linear regression; saline pump: R^2^ = 0.61, p = 0.0003, linear regression) (Figure 5D).

### Mice chronically pretreated with nicotine self-administered more nicotine than nicotine naïve mice

Following 10 days of either nicotine or saline pretreatment with an osmotic pump implant, the mice were exposed to a two bottle-choice paradigm. At the end of the tenth day of pump implantation, the pumps were removed and the mice were subjected to five repeating cycles of two bottle-choice (4 days, nicotine or vehicle) and abstinence (3 days, water). We examined whether mice that had received nicotine chronically and steadily via an osmotic pump at a dosing regimen which was sufficient to induce α4* nAChR upregulation, will choose to self-administer more nicotine and/or show a significant degree of behavioural nicotine reinforcement.

**Figure 6:**
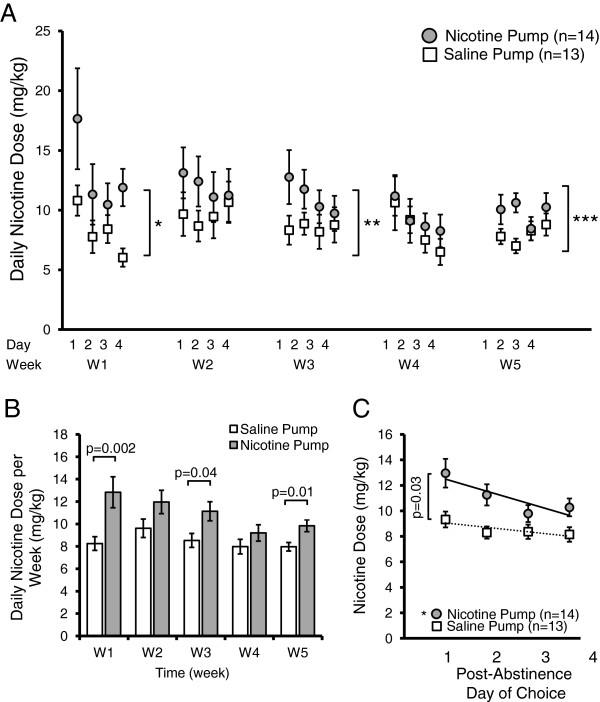
**Chronic nicotine pump mice self-administer a greater amount of nicotine and display a surge in nicotine consumption following abstinence. A)** Following 10 days of chronic drug administration via osmotic pumps (2 mg/kg/hr nicotine or saline), mice are subjected to five 7 day cycles of choice (4 days, two bottle: vehicle (0.2% saccharine) or vehicle + 200 μg/ml nicotine) followed by abstinence (3 days, single bottle roH_2_O). During choice period, nicotine primed mice consumed a greater dose of nicotine than saline pump mice (p 0.0496, pump factor, two-way mixed ANOVA). Mice implanted with a nicotine pump (n = 14) self-administered more nicotine during W1 (*p = 0.003, pump factor, two-way ANOVA), W3 (**p = 0.02, pump factor, two-way ANOVA) and W5 (***p = 0.006, pump factor, two-way ANOVA). **B)** Mean nicotine dose averaged each week of two bottle-choice self-administration showed that on a weekly basis nicotine pump mice self-administered a significantly higher level of nicotine than saline pump mice (p = 0.0496, pump factor effect, two-way mixed ANOVA; W1, p = 0.002; W3, p = 0.04; W5, p = 0.01, Bonferroni post-hoc analysis). **C)** Nicotine binge-drinking following abstinence from nicotine self-administration occurred only in the nicotine pump mice. In nicotine pump mice there was elevated nicotine consumption the day following abstinence followed by a steady decline thereafter in the next three days of choice (Solid line: R^2^ = 0.77, *p = 0.01). Saline pump mice consumed a lower dose throughout each day of the choice period (dashed line: R^2^ = 0.70, p = 0.56). Nicotine pump mice also showed a significant transient elevation in their nicotine consumption on the first day (p = 0.03, Wilcoxon rank sum test) following abstinence as compared to saline pump mice.

Figure 6 shows the drinking pattern of mice who were implanted with a nicotine pump (2 mg/kg/hr for 10 days, n = 14) or saline pump (n = 13) and then subjected to five weekly cycles of four days choice (vehicle or 200 μg/ml nicotine + vehicle; vehicle being saccharine sweetened water) followed by three days of withdrawal (roH_2_O). Nicotine pump mice (n = 14) self-administered significantly higher doses of nicotine (p = 0.0496, F(1, 23) = 4.30, pump factor effect, two-way mixed ANOVA) throughout the five week duration of the two bottle-choice experiment and reaching significance during week 1 (W1), week 3 (W3) and week 5 (W5) of the two-bottle choice experiment (W1, p = 0.002; W3, p = 0.04; W5, p = 0.01; Bonferroni post-hoc analyses) as compared to the control saline pump mice at the same respective time points (n = 13) (Figure 6A,B). Furthermore, there was a significant change in nicotine self-administration over the days of the experiment (p = 0.006, F(19, 462) = 2.04, day factor effect, two-way mixed ANOVA). However, there was no interaction effect on nicotine self-administration between the day of the experiment and the type of pump implanted (p = 0.73, F(19, 462) = 0.78, pump x day effect, two-way mixed ANOVA). We noted that nicotine pump mice showed an elevated though gradual decrease in their daily dose of nicotine (day 1- day 20: R^2^ = 0.47, p = 0.0008, linear regression) while saline pump mice maintained a steady dose of nicotine throughout the days of choice (day 1-day 20: R^2^ = 0.07, p = 0.3, linear regression).

Next, we analyzed the effects of three day nicotine deprivation on nicotine self-administration following the abstinence period by examining the trend in nicotine consumption within each of the four days of choice averaged over the five weeks (day 1, W1-W5; day 2, W1-W5; day 3, W1-W5; day 4, W1-W5) (Figure 6C). Nicotine pump mice showed a significant transient elevation in their nicotine consumption on the first day (p = 0.03, Wilcoxon rank sum test) of nicotine choice following abstinence as compared to saline pump mice, followed by a decline in nicotine consumption over the next three days (days 2–4) of the choice period (Figure 6C). This trend is observed only in animals primed with a nicotine pump (R^2^ = 0.77, p = 0.01, linear regression). Furthermore, all four days of nicotine consumption in the nicotine pump mice were elevated as compared to the saline pump mice. Saline pump mice consumed a constant dose of nicotine during each day of choice (R^2^ = 0.70, p = 0.56, linear regression).

**Figure 7:**
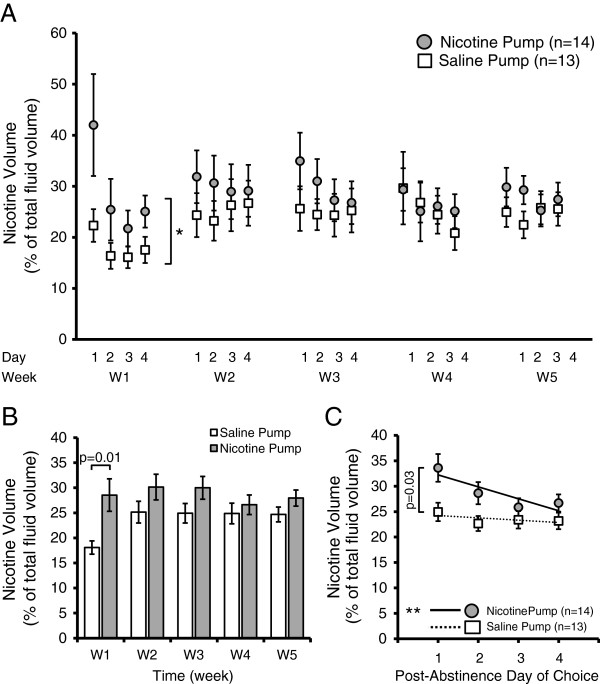
**Mice pre-exposed with chronic nicotine self-administer a greater percentage volume of their daily fluid from the nicotine containing bottle in a two-bottle choice experiment. A)** Mean nicotine volume intake as a percentage of total fluid volume during each period (days 1–4 during weeks 1–5) of two bottle-choice self-administration. Following 10 days of chronic drug administration via osmotic pumps (2 mg/kg/hr nicotine or saline), mice were subjected to five 7 day cycles of choice (4 days, two bottles: vehicle (0.2% saccharine) or vehicle + 200 μg/ml nicotine) followed by abstinence (3 days, single bottle roH_2_O). Mice primed with a nicotine containing pump self-administered more fluid from the nicotine bottle during most days of choice, though reached statistical significance only on W1 (*p = 0.0002, pump factor, two-way ANOVA; p = 0.01, Bonferroni post-hoc analysis). **B)** On a weekly basis, nicotine primed mice drink more fluid from their nicotine bottle than control saline primed mice, although significance was only achieved on W1 (p = 0.0002, pump factor, two-way ANOVA; p = 0.01, Bonferroni post-hoc analysis). **C)** Nicotine binge-drinking results from abstinence from nicotine self-administration. Percentage of daily fluid drank from the nicotine-containing bottle peaked on the first day following abstinence from nicotine self-administration and declined in subsequent days, a behaviour seen only in mice primed with a nicotine pump (day 1–4) (solid line: R^2^ = 0.76, **p = 0.01, linear regression,). Saline pump mice maintained a steady and lower percentage intake from their nicotine-containing bottle during each day of choice (dashed line: R^2^ = 0.36, *p = 0.19, linear regression). Furthermore, nicotine-pump mice self-administered significantly more nicotine compared to saline-pump mice on the first day following abstinence (p = 0.03, two sample t-test).

Mice control their daily fluid intake closely; during periods of choice their total fluid volume is composed of fluid from each of two bottles: vehicle or 200 μg/ml nicotine + vehicle. We examined the percentage of their total fluid intake that is allocated to the bottle which contains nicotine (Figure 7). During week 1 (W1), the mice primed with a nicotine pump drank a significantly higher percentage of total fluid from the nicotine bottle (p = 0.0002, F(1, 487) = 14.2, pump factor effect, two-way ANOVA; p = 0.01 for W1, Bonferroni post-hoc analysis) as compared to the bottle containing only saccharine sweetened water (Figure 7B). During subsequent weeks (W2-W4), the percentage of daily fluid consumed from the nicotine bottle was not significantly different between the nicotine and saline pump animals. There was neither a significant effect of days of the experiment (p = 0.36, F(19, 487) = 1.08, day factor effect, two-way ANOVA) nor an interaction between pump and day effect on the percentage volume of nicotine consumed (p = 0.92, F(19, 487) = 0.57, pump x day factor effect, two-way ANOVA). When we examined their nicotine volume as a percent of total fluid volume across all days of choice, we found that, overall, mice primed with nicotine pumps drank a significantly larger portion of their daily fluid intake from the nicotine-containing bottle as compared to the saline pump mice (p = 0.002, Wilcoxon rank sum test) (data not shown).

Within each week, the nicotine pump mice showed a significant initial surge in fluid consumption from the nicotine-containing bottle on day one (p = 0.03, t-test) as compared to the saline pump mice on day one and followed by a steady decline during the next three days in their nicotine consumption as a percentage of total fluid volume (R^2^ = 0.76, p = 0.01, linear regression). The saline pump mice consumed a steady (R^2^ = 0.36, p = 0.19, linear regression) and lower percentage of their daily fluid volume from both bottles across all four days of choice as compared to nicotine pump mice. These results indicate that three days of abstinence resulted in binge drinking of nicotine immediately upon resumption of the nicotine and water choice bottles.

**Figure 8:**
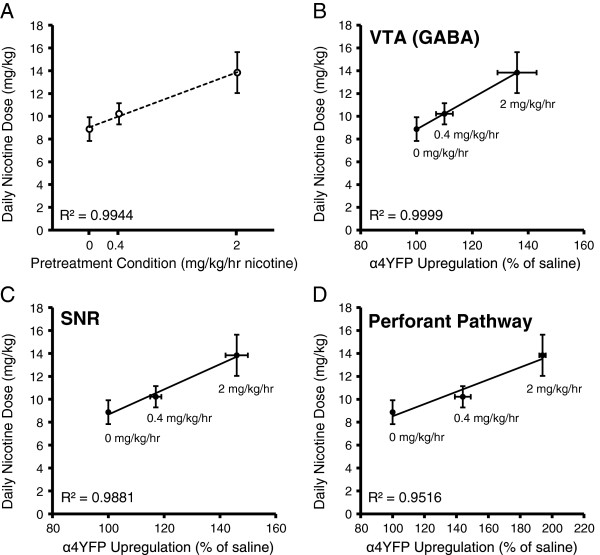
Pretreatement with chronic nicotine and α4* nAChR upregulation correlate with nicotine self-administration. Mice implanted with osmotic pumps (saline (n = 3), 0.4 mg/kg/hr (n = 5) or 2 mg/kg/hr nicotine (n = 4)) for 10 days were subjected to two-bottle choice nicotine self-administration for 5 days following pump removal. **A)** Pretreatment with nicotine correlates with mean daily dose of self-administered nicotine (R^2^ = 0.994, p = 0.01, linear regression). Osmotic pump treatment with 0.4 or 2 mg/kg/hr nicotine for 10 days results in a progressively greater amount of α4YFP nAChR upregulation as compared to treatment with 0 mg/kg/hr (% of saline). The α4YFP nAChR upregulation data for the three doses were taken from Nashmi et al. [7] and show that oral self-administration strongly correlates with upregulation in GABAergic neurons of the VTA **(B)** (R^2^ = 0.9999), GABAergic neurons of the substantia nigra pars reticulata (SNR) **(C)** (R^2^ = 0.988) and the medial perforant pathway of the hippocampus **(D)** (R^2^ = 0.952).

To examine whether nicotine self-administration correlates with nAChR upregulation we performed an experiment involving three groups of mice chronically exposed to different concentrations of nicotine via osmotic pumps: 1) saline (0 mg/kg/hr nicotine), 2) 0.4 mg/kg/hr nicotine, and 3) 2 mg/kg/hr nicotine. Following the 10 days of saline or nicotine pump administration, the pumps were removed and mice underwent 5 days of two bottle-choice of oral nicotine self-administration. There was a strong linear positive correlation of the dose of nicotine administered through the pumps and the degree of oral nicotine self-administration (R^2^ = 0.994) (Figure 8A). We then examined whether oral nicotine self-administration in the three groups of mice correlated with α4YFP nAChR subunit upregulation in three different brain regions: the medial perforant path of the hippocampus, GABAergic neurons of the VTA and GABAergic neurons of the substantia nigra (SNR). Since we closely replicated nicotine induced expression changes of α4YFP nAChR subunits at 2 mg/kg/hr to our previous study (Table 1) [7], we used the α4YFP expression data for saline, 0.4 mg/kg/hr nicotine and 2 mg/kg/hr nicotine from Nashmi et al. [7] to correlate with oral nicotine self-administration in this study from mice undergone the same dosage of nicotine pump administration (0, 0.4 and 2 mg/kg/hr for 10 days) (Figure 8B-D). We found that oral nicotine self-administration displayed a strong positive correlation with α4YFP nAChR subunit upregulation in all three brain regions with the highest correlation in GABAergic neurons of the VTA (VTA: R^2^ = 0.9999; SNR: R^2^ = 0.988; medial perforant path: R^2^ = 0.952). These results provide supporting evidence suggesting that α4* nAChR upregulation may contribute to nicotine self-administration.

## Discussion

In this study we examined whether nicotinic receptor upregulation correlates with oral self-administration of nicotine by comparing nicotine drinking behaviour in mice pretreated with chronic nicotine via an osmotic pump as compared to chronic saline controls. Ten days of chronic nicotine treatment (2 mg/kg/hr) via osmotic pumps resulted in significant upregulation of α4 nAChR subunits in the hippocampal medial perforant path and GABAergic neurons of the VTA in α4YFP knock-in mice. The upregulation was cell type specific since there was neither upregulation in the medial habenula nor on dopaminergic neurons in the VTA. Chronic nicotine pretreated mice consumed a significantly greater daily dose of nicotine as compared to mice pretreated with saline and varying doses of chronic nicotine pretreatment linearly correlated with both the degree of nicotine self-administration as well as nAChR upregulation. Furthermore, following three days of abstinence the nicotine pretreated group of mice consumed significantly elevated nicotine levels during the first day of choice. These results are consistent with the notion that nicotinic receptor upregulation may contribute to nicotine dependence.

### α4* nAChR upregulation is brain region and cell type specific

We show that quantitative fluorescence imaging provides a consistent and reliable means of examining cell type and brain region specific nAChR upregulation. Utilizing the protocol described by Renda and Nashmi [32], we have reproduced quantitatively very similar levels of nicotinic receptor upregulation in specific neuronal cell types and brain regions as first described by Nashmi and colleagues [7].

The medial perforant path of the hippocampus shows improvement of long term potentiation induction following application of chronic nicotine [7], which may account for improvements in learning and memory seen in smokers and chronically exposed mice [36]. The medial habenula is related to nicotine aversion and plays a role in withdrawal from chronic nicotine [22, 37]. Both the medial perforant path and the medial habenula express α4* nAChRs, although only the medial perforant path shows nAChR upregulation in response to chronic nicotine, while the expression of α4* nAChRs in the medial habenula is unchanged. The VTA is strongly linked to nicotine addiction [4, 15, 38, 39]. Although both dopaminergic and GABAergic neurons of the VTA express α4* nAChRs, upregulation is restricted to GABAergic neurons. However, the increase in nicotinic receptors confers an increase in GABAergic inhibition of dopaminergic activity which subsequently decreases the reward output of the VTA. This is hypothesized to account for the addictive behaviours of craving and tolerance in nicotine addicts [7]. Our fluorescence imaging results suggest that mice pretreated with nicotine via osmotic pumps may be tolerant to nicotine due to GABAergic specific nAChR upregulation in the VTA. Therefore, it stands to reason that if upregulation plays a role in nicotine dependence and self-administration these mice would need to intake a larger dose of nicotine, compared to nicotine naïve mice, to experience reinforcement.

### Chronic nicotine pretreatment enhances nicotine self-administration

We sought to address the question of whether chronic nicotine pre-exposure can influence the choice to self-administer nicotine. By first showing that chronic nicotine administration via osmotic pumps at 2 mg/kg/hr is sufficient to consistently upregulate α4* nAChRs in the hippocampus and VTA, we placed pretreated mice into a two bottle-choice self-administration paradigm. Since we have coupled drug administration with fluid intake, providing the animal with a choice is crucial in order to investigate the more subtle behaviours contributing to addiction and making accurate inferences about how molecular changes following nicotine exposure influence the choice to self-administer nicotine.

Mice pretreated with chronic nicotine self-administered a greater daily dose of nicotine throughout the 20 days of exposure (Figure 6A). Self-administration of nicotine is absent in both α4 and β2 KO mice but selective re-expression of the knocked-out subunit (either α4 or β2) in the VTA of KO mice re-establishes nicotine self-administration [5, 15], which forges a strong link between the activity of these subunits and nicotine dependence. Our data suggest that in addition to α4β2 nAChRs governing nicotine self-administration, the level of α4* nAChR expression can influence the magnitude of a mouse’s propensity towards nicotine self-administration. At this point our data is consistent with the hypothesis that nicotinic receptor upregulation is contributing to the heightened self-administration of nicotine in mice. However, to unequivocally test this hypothesis further experiments would need to be performed to knock-down nicotinic receptor expression during chronic pretreatment of nicotine to determine whether it is α4* nAChR upregulation or an ancillary effect of nicotine that is essential for nicotine dependent behaviour. Molecular and genetic tools such as shRNA, knock-out mice and conditional transgenic mice can shed light onto this question. One potential caveat is that oral nicotine self-administration may be driven by different factors as compared to i.v. or intra-VTA self-administration. Furthermore, it is not yet known whether deletion of α4 or β2 subunits would have the same effect of attenuating oral self-administration of nicotine as had been found for i.v. and intra-VTA self-administration [4, 5, 15] though deletion of the β2 nAChR gene did not appear to alter forced nicotine drinking in mice [40]. Therefore, future studies using nicotine two bottle-choice oral self-administration experiments with α4 and β2 KO mice would need to be performed in order to further elucidate the role of these specific subunits in nicotine self-administration.

### Mice pretreated with chronic nicotine exhibit abstinence induced binge-drinking

The data presented in Figure 6C showed that abstinence from nicotine for three days following a choice period results in significantly elevated nicotine self-administration on the first day following abstinence and a gradual decline over the next three days of choice in nicotine pump mice, a behaviour which was absent in saline pump mice. This elevation in drinking levels may be due to binge-drinking as a means to relieve withdrawal symptoms. This is consistent with the results of Damaj and colleagues [21], who showed that somatic withdrawal signs in mice peak between 2 and 3 days following removal of osmotic pumps chronically infusing nicotine at 48 mg/kg/day over 15 days. Withdrawal can be divided into three broad categories: physical, affective and cognitive withdrawals. Each yields different symptoms, although the cognitive deficits linked to nicotine withdrawal are thought to be mediated by α4β2 nAChRs [24–27] and produce strong negative reinforcement which decreases reward signals [41]. Abstinence causes a drug-binge once nicotine is reintroduced to the animal [12, 42]. We show that nicotine-primed mice ingest a significantly higher nicotine dose on the first day following abstinence, compared to control mice, and that these values decrease over subsequent days of choice. Binging is absent in the control mice also provided with the two bottle-choice. We propose that cognitive deficits and affective and physical discomfort elicited by abstinence from nicotine decrease in severity as upregulation declines following removal of forced nicotine delivery via pump [41, 43], which induces maximal nAChR upregulation. Nicotine craving, linked to nAChR upregulation, may be exacerbated by the negative reinforcement of withdrawal, causing the mice to consume more nicotine immediately following abstinence.

A study by Hillario and colleagues [44] showed that there was increased brain reward function in mice with repeated cycles of withdrawal, which initially appears to contradict our results of the declining levels of enhanced nicotine self-administration of nicotine pump mice over time. In the study by Hilario et al. they continuously infused nicotine at 24 mg/kg/day over 14 days followed by 4 days of single nicotine injections (0.5 mg/kg) and 4 days of withdrawal. This cycle was repeated three times so a total of 42 days of chronic nicotine was delivered by osmotic pumps before the third withdrawal period and the last nicotine pump delivery was only 4 days prior to the start of the withdrawal period. The repeated pump delivery of nicotine showed the greatest level of nAChR upregulation by the third cycle of nicotine administration and resulted in elevated reward brain function in the nicotine pump mice. Although our study also had repeated periods of withdrawal, the experimental paradigm of Hilario et al. differed markedly from our study, in which only one continuous application of 10 days of nicotine (2 mg/kg/hr) was delivered via osmotic pumps and that following the third cycle of withdrawal in our study oral nicotine self-administration was measured 21 days following the last day of mini-pump nicotine administration. Therefore, both studies are consistent in that for our study nicotinic receptor upregulation would be greatest during the first week, during which we saw the largest degree of nicotine self-administration, and receptor levels would decline over time proportional to the level of self-administration, which may explain the diminished but still heightened nicotine self-administration in nicotine pump mice as compared to the saline pump control mice over time. Hilario and colleagues also showed the importance of nAChR upregulation, which peaked during the third cycle of withdrawal and correlated with enhanced brain reward function. Therefore, both studies are consistent, albeit using different methods, in showing the significance of nAChR upregulation in behaviours related to nicotine dependence.

### Role of nAChR upregulation in nicotine self-administration

Pretreating with chronic nicotine is a novel feature of self-administration paradigms. Cocaine had been used for a similar rationale to train the mice to expect a drug-induced reward in order to study addictive behaviours [45]. We have adapted a similar principle, but unlike pre-exposure with a drug like cocaine, which would instill an addiction via different molecular pathways, we have primed our mice with nicotine and have therefore elicited a biochemical change that is nicotine-dependent and can implicate nicotinic receptor upregulation as a contributor of elevated self-administration observed in pretreated mice.

Using three different concentrations of chronic nicotine pretreatment (0, 0.4 and 2 mg/kg/hr nicotine) via osmotic pumps we have shown a strong positive linear correlation of nicotine dose and oral nicotine self-administration (Figure 8A). Moreover, these three different doses of nicotine pretreatment resulted in a strong positive correlation between α4YFP nAChR upregulation and oral nicotine self-administration in three different brain regions (hippocampal medial perforant path, VTA GABAergic neurons, SNR neurons) (Figure 8B-D). Interestingly, oral nicotine self-administration showed the highest positive correlation with α4YFP nAChR upregulation in GABAergic neurons of the VTA, a brain region important for reward and strongly implicated in addiction of drugs of abuse.

The data in Figure 6A show that the nicotine primed mice are gradually decreasing their daily nicotine dose, suggesting that they are unable to administer a large enough dose to satiate the craving of such a high receptor level instilled by the pumps without succumbing to the negative side-effects of a nicotine overdose. We speculate that the nicotine the pretreated mice ingest each day may be providing the maximum satisfaction to withdrawal ratio while their receptors normalize to a steady level consistent with their maximum daily nicotine consumption.

Conversely, the saline pump mice have gradually increased the amount of fluid consumed from the nicotine-containing bottle over the course of the experiment in order to maintain a steady daily dose of nicotine, which they accomplish despite a weight increase – suggesting that they are experiencing consistent reinforcement, befitting what is known as the Boundary Model [46]. Human smokers are subdivided into two groups: nicotine-addicted heavy smokers and chippers – light smokers who show few signs of addiction. Both groups smoke regularly and in similar patterns, although light smokers do not become tolerant, they maintain a constant, unchanging daily dose. Heavy smokers, however, become tolerant to the effects of nicotine over time and subsequently increase their daily nicotine dose. Mice behave similarly; they are habitual in that they actively maintain a constant dose of nicotine by adjusting their consumption from the nicotine-containing bottle over time, suggesting that they are satisfied without becoming tolerant and having to increase their daily dose. We argue that both groups of mice are exhibiting signs of dependent behaviour and that it is possible they are both converging on a similar satisfactory nicotine dose. Mice not pretreated with chronic nicotine, who have unaffected levels of nAChRs, maintain a consistent daily nicotine dose similar to light smokers and do not exhibit tolerance. Conversely, the forced nicotine delivery by an osmotic pump, secreting a five-fold higher dose of nicotine compared to peak self-administration doses, may yield a level of upregulation which the mice are incapable of maintaining with oral self-administration of nicotine. Still, we are able to correlate nicotinic receptor upregulation, caused by forced pretreatment with an osmotic pump, with an increase in the magnitude of nicotine self-administration.

Another potential contributing factor to the significantly elevated nicotine drinking behavior of the nicotine primed mice is the issue of receptor desensitization. As proposed by Dani and Heinemann [47] a possible explanation of increased nicotine intake for smokers is that the abnormally high synaptic activity that would be brought about by upregulated receptors in the non-rewarding cholinergic synapses could contribute to withdrawal symptoms. The consumption of nicotine would desensitize the additional upregulated nAChRs down to normal nicotinic activity and thus, would relieve withdrawal symptoms. The potential reason that nicotine self-administration is greatest for the nicotine pump mice the first week following pump removal and slowly declined nicotine consumption in subsequent weeks is due to the fact that receptor upregulation is likely greatest at the first week following pump administration when nicotine administration was forced at a level five times greater than possible than possible with the self-administered dose. Over time the receptors would come back closer to normal or saline pump levels due to the inability of oral self-administration of nicotine to out-pace the constant high concentration of nicotine delivery via pumps. Thus, we speculate that nicotine pretreated mice would need to consume less nicotine over time since there are less upregulated receptors needing to be desensitized back to their normal level of activity. Continuous chronic delivery of nicotine has been shown to result in a peak expression of high affinity nAChRs by seven days and fall back to baseline control levels by eight days following removal of nicotine exposure [43]. This is consistent with the time course of the oral nicotine consumption in our study; however, the oral nicotine consumption would maintain some degree of receptor upregulation to perpetuate elevated oral nicotine consumption.

## Conclusions

This study demonstrates that chronic pre-exposure to nicotine at a dosing regimen that results in maximal α4* nAChR upregulation results in significantly elevated oral nicotine self-administration in a two-bottle choice paradigm in mice. We also demonstrate that chronic nicotine pretreated mice exhibit heightened binge drinking immediately following a period of nicotine abstinence. To conclusively demonstrate causality that nAChR upregulation contributes to nicotine addiction would require further studies using molecular strategies targeting the knock-down of α4 nAChR subunits and studying the effect on nicotine self-administration.

## Abbreviations

ACh: Acetylcholine
nAChRs: Nicotinic acetylcholine receptors
roH20: Reverse osmosis water
SNC: Substantia nigra pars compacta
SNR: Substantia nigra pars reticulata
VTA: Ventral tegmental area.
